# Hypothalamic Cavernous Malformation: Surgical Technique and Literature Review

**DOI:** 10.7759/cureus.21511

**Published:** 2022-01-23

**Authors:** Nataly Alvear-Quito, Alejandro Ceja-Espinosa, Juan P Navarro-Garcia de Llano, Aurelio Ponce-Ayala, Edgar Nathal

**Affiliations:** 1 Neurosurgery, Instituto Nacional de Neurología y Neurocirugía Manuel Velasco Suárez, Mexico City, MEX

**Keywords:** cavernous malformation, cavernous angioma, hypothalamic cavernoma, hemangioma, hypothalamus

## Abstract

Hypothalamic cavernous malformation (HCM) is rare, and to our knowledge, there are only 28 cases reported in the literature.

An 18-year-old male presented two years ago with a severe headache followed by right eye blindness. Following imaging studies, a bleeding hypothalamic cavernoma was discovered together with another incidental cavernoma in the brain. We sustained the diagnosis of cavernomatosis, and conservative treatment was indicated. A year later, he presented severe headache and vomit; for this reason, the patient underwent a new MRI which showed a new bleeding episode of the HCM lesion. We carried out an endocrinological assessment, and microsurgical resection was recommended. Although visual impairment persisted as expected in the postoperative period, he showed good clinical recovery overall.

Hypothalamic location of a cavernous malformation is infrequent, accounting for only 1% or less of these lesions, and are known to cause a variety of symptoms inducing headache, visual disturbance, and less frequently, hypothalamus dysfunction. Surgical intervention can be considered after a second symptomatic bleed, always assessing the risk of non-favorable postsurgical outcomes against the intrinsic risk that these malformations imply.

Case reports like this are essential to reach a consensus towards the best treatment option for HCM.

## Introduction

Hypothalamic cavernous malformation (HCM) is rare, accounting for only 1% or less among cavernoma locations [1]. To our knowledge, only 28 cases in the literature have been reported to date [2]. Typically, cavernomas in this location present with headache, visual, and endocrinological disturbances. On imaging, detecting these malformations is difficult due to their similarity with other low-flow vascular abnormalities [3].

According to the literature, the general rate of hemorrhage for cavernous malformations reaches 3.1% per year, and it is well known that the risk for second bleeding if untreated increases dramatically [4]. A consensus is needed to establish guidelines for the management of this rare entity.

This article describes case number 29, discusses our rationale for surgical treatment, the patient’s outcome, and briefly reviews the literature regarding this rare pathology.

## Case presentation

**Figure 1:**
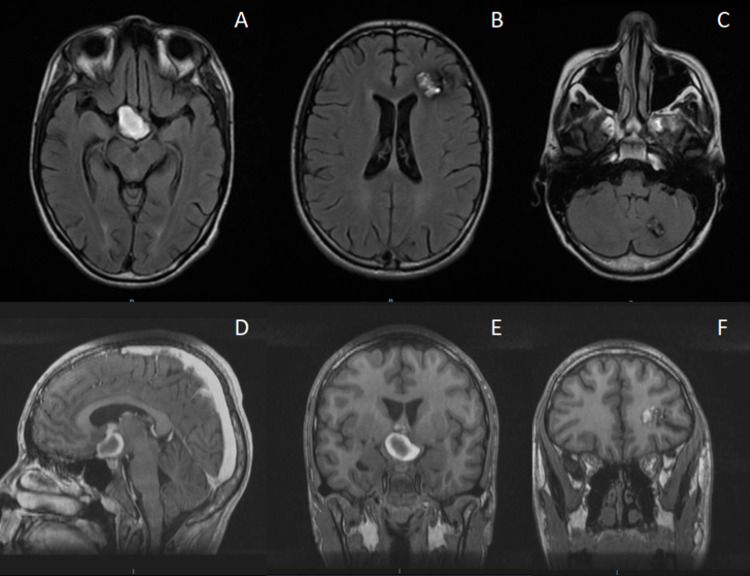
Imaging studies two years before coming to us. A-C, axial T2-FLAIR MRI shows multiple cavernous malformations, hypothalamic, cerebellar, and frontal lesions (left to right). D-E, hypothalamic cavernous malformation in different T1-weighted MRI projections, sagittal and coronal (left to right). F, frontal cavernous malformation on a coronal T1-weighted MRI

We present the case of an 18-year-old male patient without any relevant past medical history. Two years before admission, he presented with a 10/10 headache on the visual analog scale, followed by right eye blindness. For these reasons, we performed a gadolinium contrast MRI of the head, which showed hyperintense frontal, suprasellar, and cerebellar lesions, with classical “popcorn” appearance compatible with cavernomatosis (Figure 1). At that time, conservative treatment was decided by another neurosurgeon.

**Figure 2:**
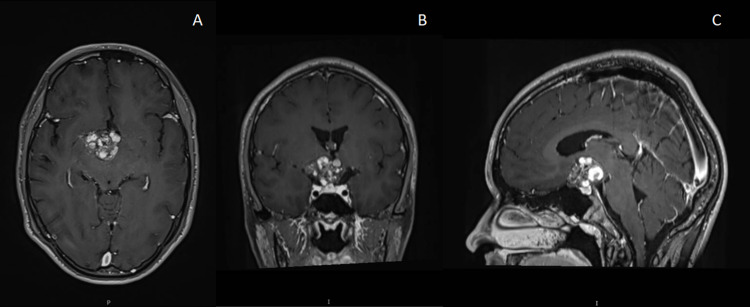
T1-weighted MRI at the time of arrival to our Institution. Rebleeding of the hypothalamic cavernous malformation is appreciated in different projections. A, Axial. B, Coronal. C, Sagittal.

One year later, he presented to the emergency department (ED) of our institution with an intense generalized headache of sudden onset, as well as vomiting. At arrival, the patient was awake, oriented, cooperative, without speech deficit. The right eye was amaurotic and visual acuity was 20/40 in the left eye, presenting temporal hemianopsia; the right pupil was dilated, non-reactive to light, with conserved consensual reflex. There were no left pupil alterations, abnormal eye movements, or pathological changes at fundus; gait was not assessable. The rest of the neurological physical examination was normal. A new head MRI showed the same lesions previously mentioned, with an increase in the dimensions of the suprasellar lesion, compatible with cavernous malformation rebleeding (Figure 2). Endocrinological and neuro-ophthalmological examinations were made, finding subclinical hypothyroidism and bilateral optic nerve atrophy, respectively, with a limited prognosis for visual function.

**Figure 3:**
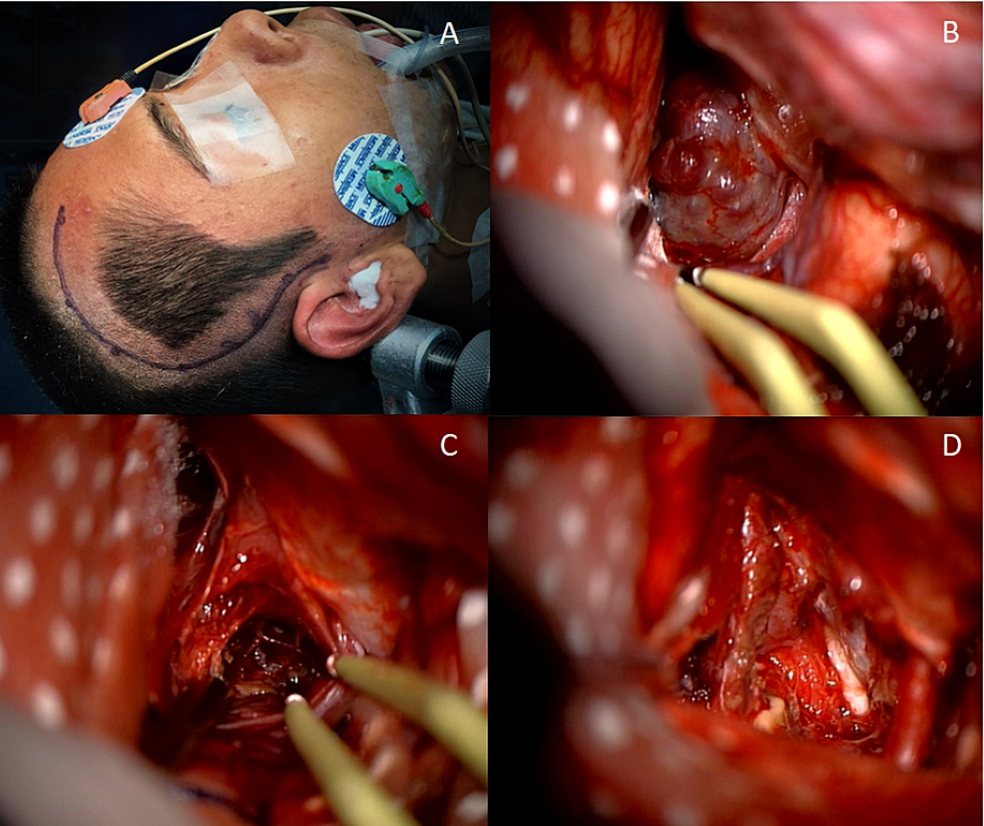
Microsurgical procedure. A, Right pterional approach. B, Hypothalamic cavernous malformation is observed. C, Opening of the cavernous malformation’s surface. D, Final view after gross total resection.

**Figure 4:**
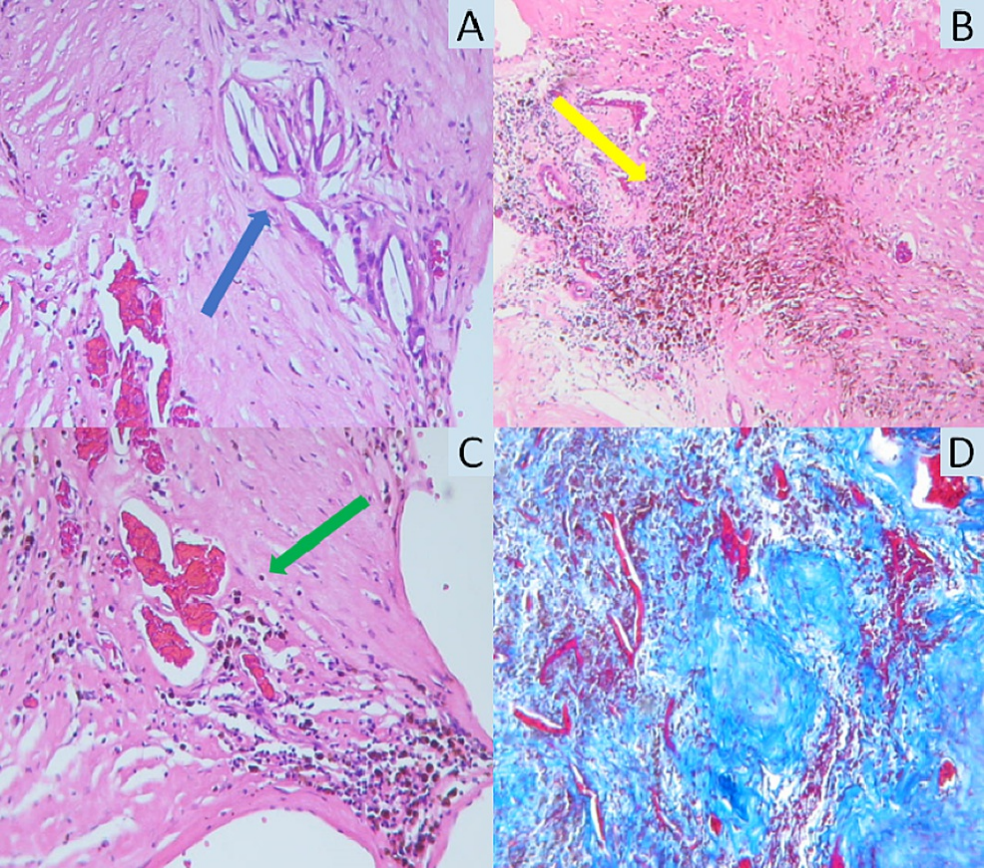
Histology. A-C, H&E stain where we can appreciate cholesterol crystals (blue arrow), hemosiderin (yellow arrow), and abnormal vessels (green arrow). D, Masson’s trichrome stain showing fibrosis surrounding the blood vessels.

It was initiated management with levothyroxine, and the patient underwent surgery through a left pterional approach for a gross total resection. The patient was placed supine, and a Yasargil-type incision extending below the root of the zygoma was done (Figure 3A); the temporal muscle was detached with curettage followed by a single burr hole in the most caudal portion of the approach, then, a frontotemporal craniotomy was completed. Durotomy was made in a semilunar fashion with a rostral base, and dissection of the Sylvian fissure was carried out. Suprachiasmatic, bilateral carotid, and optic cisterns were drained, leaving the cavernous malformation visible on the pial surface (Figure 3B). The cavernous malformation was removed, preserving the pituitary stalk and hypothalamus (Figure 3C,D). The obtained mass was sent to pathology, confirming the diagnosis (Figure 4).

**Figure 5:**
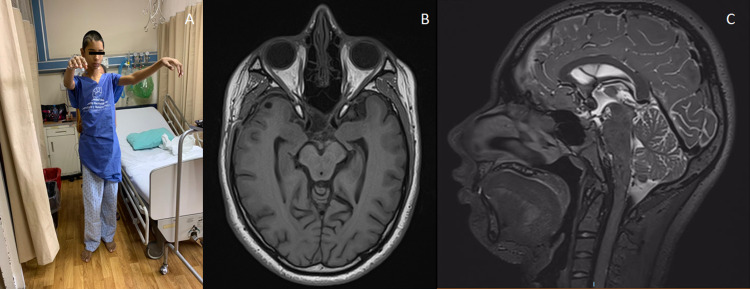
Fourth day post-op. A, Patient showing good surgical recovery. B-C, Axial MRI T1-weighted and Sagittal T2.weighted images showing complete cavernous malformation’s resection.

In the immediate postoperative period, the patient presented diabetes insipidus, managed with nasal desmopressin showing an adequate clinical response. On the fourth post-op day, the patient showed good clinical recovery (Figure 5A) with the persistence of the visual deficit. A postoperative MRI confirmed a total resection of the malformation (Figure 5B,C). The other cavernomas were not treated. The patient was the first confirmed case in his family; a year later, his aunt was also detected with similar lesions, documenting the diagnosis of familial cavernomatosis.

## Discussion

Cavernous malformations are collections of endothelium-lined sinusoids without intervening cerebral parenchyma and macroscopically have the aspect of a mulberry [3]. They are low-flow vascular lesions that are typically angiographically occult [4]. Their prevalence across several reports ranges from 0.4% to 0.6% of the population and represents the second most common vascular anomaly after high-flow arteriovenous malformations [5]. The hypothalamic location of a cavernoma is rare, accounting for only 1% or less [1]. They can appear anywhere in the central nervous system, as a single lesion (sporadic cavernoma) or associated with developmental venous anomalies. It is also possible to find multiple lesions in the familial cases due to an autosomal dominant inherent mutation related to CCM1, CCM2, and CCM3 genes [6]. Nonetheless, cavernomas can also occur after radiotherapy, stereotactic biopsy, and central nervous system infections [7]. The clinical expression varies depending on the location; it has been noted that lesions in the third ventricle boundaries and hypothalamic area induce headache, visual disturbance, memory loss, and inappropriate secretion of antidiuretic hormone syndrome [4, 8]. 

The gold standard for radiological diagnosis is magnetic resonance. In 1994, Zabramski classified these lesions into four types according to their bleeding stage [9]. The classical appearance of these lesions is that of areas of mixed-signal intensity with a hypointense rim described as a "popcorn" appearance (Zabramski Type 2). The differential diagnosis for lesions in the hypothalamic region includes hemorrhagic neoplasms, gliomas, hypothalamic hamartomas, colloid cysts, craniopharyngiomas, and germinomas [3, 9]. Despite decades of neurosurgical experience in this field, evidence supporting surgical resection of cavernous malformations of the brain remains conflicting. Current treatment options include clinical surveillance, microsurgical excision, or radiosurgery [10]. Surgical intervention can be considered mainly following a second symptomatic bleed after evaluating the risk of postoperative deficits versus a possible aggressive course in the long term, especially those located in eloquent or profound zones [11]. Lesions of the hypothalamus present an operative challenge because of their deep location and eloquence. Surgical approaches include anterolateral approaches such as pterional, supraorbital, or orbitozygomatic and midline approaches (transbasal subfrontal or transbasal interhemispheric) [4]. In this particular case, a pterional approach was selected because of the lesion's size and its relationship with the pia surface, making it visible since the early phase of surgery.

Even though the initial approach was conservative due to the deep location of the cavernoma, the patient had neurological worsening and documented growth of the lesion, compromising visual and endocrinological functions. For this reason, surgery was recommended and performed through a right anterolateral approach without any significant postoperative neurological deficit. At follow-up, the patient presented with an mRS of 1, which is an excellent outcome for this type of lesion, with a total resection demonstrated by the MRI six months after surgery.

## Conclusions

Hypothalamic cavernomas are rare, and even though we gain more experience in treating these cases every year, there is still a lack of consensus towards the best treatment, surgical or conservative. Then, reports like this are essential to have a more realistic scenario about morbidity and mortality after selecting a treatment modality. We consider that in this case, there was appropriate management based on the patient's background, age, clinical findings, and potential risk for complications. A complete presurgical assessment, including endocrinological and ophthalmological evaluations, is necessary, considering the importance of the structures at stake.

We encourage other authors to report their experiences and results with surgery and different surgical approaches as well as those cases with conservative or radiosurgery treatments to establish a consensus based on accumulative experience.
